# Supplementary material for: The Incidence of Adverse Events in Adults Undergoing Procedural Sedation with Propofol Administered by Non-Anesthetists: A Systematic Review and Meta-Analysis
Source: Diagnostics (Basel). 2025 May 14;15(10):1234. doi: 10.3390/diagnostics15101234 (PMC12110594; doi:10.3390/diagnostics15101234)
Supplement: Supplementary file 1 [file diagnostics-15-01234-s001.zip › S1.pdf]

Appendix 1. Search strategy

Pubmed

((esophagogastroduodenoscopy OR endoscopy digestive system[mesh] OR Digestive System Endoscopic Surgery OR gastrointestinal endoscopy[ti] OR endoscopy, gastrointestinal[mesh] OR colonoscopy[mesh] OR colonoscopy OR colonoscopic surgery OR Colonoscopic Surgical Procedure OR Endoscopic retrograde cholangiopancreatography OR Cholangiopancreatography, Endoscopic Retrograde[Mesh] OR ERCP OR endosonography OR endoscopy echo OR Endoscopic Ultrasonography OR endoscopic ultrasound OR Endoscopic Gastrointestinal Surgery AND (2010:2023[pdat])) AND (deep sedation[mesh] OR deep sedation[ti] OR conscious sedation[mesh] OR conscious sedation[ti] OR sedoanalgesia OR non-anesthesiologist sedation OR non-anesthesiologist propofol OR naas[ti] OR naap[ti] OR sedoanalgesia OR sedation[ti] AND (2010:2023[pdat]))) AND (nurse OR non-anesthesiologist OR endoscopist OR gastroenterologist)

Embase

('nurse'/exp OR nurse OR 'non anesthesiologist' OR 'endoscopist'/exp OR endoscopist OR 'gastroenterologist'/exp OR gastroenterologist) AND ('deep sedation'/exp OR 'deep sedation' OR (deep AND ('sedation'/exp OR sedation)) OR 'conscious sedation'/exp OR 'conscious sedation' OR (conscious AND ('sedation'/exp OR sedation)) OR 'non-anesthesiologist sedation' OR ('non anesthesiologist' AND ('sedation'/exp OR sedation)) OR 'non-anesthesiologist propofol' OR ('non anesthesiologist' AND ('propofol'/exp OR propofol)) OR naas OR naap OR 'sedoanalgesia'/exp OR sedoanalgesia OR 'sedation'/exp OR sedation) AND ('esophagogastroduodenoscopy'/exp OR esophagogastroduodenoscopy OR 'endoscopy digestive system'/exp OR 'endoscopy digestive system' OR (('endoscopy'/exp OR endoscopy) AND digestive AND system) OR 'digestive system endoscopic surgery' OR (digestive AND system AND endoscopic AND ('surgery'/exp OR surgery)) OR 'gastrointestinal endoscopy'/exp OR 'gastrointestinal endoscopy' OR (gastrointestinal AND ('endoscopy'/exp OR endoscopy)) OR 'colonoscopy'/exp OR colonoscopy OR 'colonoscopic surgery' OR (colonoscopic AND ('surgery'/exp OR surgery)) OR 'colonoscopic surgical procedure' OR (colonoscopic AND surgical AND ('procedure'/exp OR procedure)) OR 'endoscopic retrograde cholangiopancreatography'/exp OR 'endoscopic retrograde cholangiopancreatography' OR (endoscopic AND retrograde AND ('cholangiopancreatography'/exp OR cholangiopancreatography)) OR 'ercp'/exp OR ercp OR 'endosonography'/exp OR endosonography OR 'endoscopy echo' OR (('endoscopy'/exp OR endoscopy) AND ('echo'/exp OR echo)) OR 'endoscopic ultrasonography'/exp OR 'endoscopic ultrasonography' OR (endoscopic AND ('ultrasonography'/exp OR ultrasonography)) OR 'endoscopic ultrasound'/exp OR 'endoscopic ultrasound' OR (endoscopic AND ('ultrasound'/exp OR ultrasound)) OR 'endoscopic gastrointestinal surgery' OR (endoscopic AND gastrointestinal AND ('surgery'/exp OR surgery)))

Cochrane

(esophagogastroduodenoscopy OR endoscopy digestive system OR Digestive System Endoscopic Surgery OR gastrointestinal endoscopy OR endoscopy, gastrointestinal OR colonoscopy OR colonoscopic surgery OR Colonoscopic Surgical Procedure OR Endoscopic retrograde cholangiopancreatography OR Cholangiopancreatography, Endoscopic Retrograde OR ERCP OR endosonography OR endoscopy echo OR Endoscopic Ultrasonography OR endoscopic ultrasound OR Endoscopic Gastrointestinal Surgery) AND (deep sedation OR conscious sedation OR sedoanalgesia OR non-anesthesiologist sedation OR non-anesthesiologist propofol OR naas OR naap OR sedation) AND (nurse OR non-anesthesiologist OR endoscopist OR gastroenterologist) in Title Abstract Keyword - (Word variations have been searched)
