# Supplementary material for: The Incidence of Adverse Events in Adults Undergoing Procedural Sedation with Propofol Administered by Non-Anesthetists: A Systematic Review and Meta-Analysis
Source: Diagnostics (Basel). 2025 May 14;15(10):1234. doi: 10.3390/diagnostics15101234 (PMC12110594; doi:10.3390/diagnostics15101234)
Supplement: Supplementary file 1 [file diagnostics-15-01234-s001.zip › S2.pdf]

Appendix 2. Articles included in the meta-analysis

1. Frieling T, Heise J, Kreysel C, et al. Sedation-associated complications in endoscopy - Prospective multicentre survey of 191142 patients. Article. Zeitschrift fur Gastroenterologie. 2013; 51:568-572.

2. Ruiz-Curiel R, Bonilla-H Y, Baptista A, et al. Sedation with propofol in digestive endoscopy administered by gastroenterologists. Experience in a Venezuelan hospital. Article. Revista Espanola de Enfermedades Digestivas. 2018; 110:246-249

3. Sato M, Horiuchi A, Tamaki M, et al. Safety and Effectiveness of Nurse-Administered Propofol Sedation in Outpatients Undergoing Gastrointestinal Endoscopy. Clin Gastroenterol Hepatol. 2019; 17:1098-1104.

4. Ooi M, Thomson A. Morbidity and mortality of endoscopist-directed nurse-administered propofol sedation (EDNAPS) in a tertiary referral center. Endosc Int Open. 2015;3: E393-7.

5. Akyuz U, Pata C, Senkal V, et a. Is propofol sedation with midazolam induction safe during endoscopic procedures without anesthesiologist? Hepatogastroenterology. 2010; 57:685-7.

6. García-Suárez C, López-Rosés L, Olivencia P, et al. Sedation with propofol controlled by endoscopists during percutaneous endoscopic gastrostomy. Rev Esp Enferm Dig. 2010; 102:249-56.

7. Poincloux L, Laquière A, Bazin JE, et al. A randomized controlled trial of endoscopist vs. anaesthetist-administered sedation for colonoscopy. Dig Liver Dis. 2011; 43:553-8.

8. Repici A, Pagano N, Hassan C, et al. Balanced propofol sedation administered by nonanesthesiologists: The first Italian experience. World J Gastroenterol. 201; 17:3818-23.

9. Lee CK, Lee SH, Chung IK, et al. Balanced propofol sedation for therapeutic GI endoscopic procedures: a prospective, randomized study. Gastrointest Endosc. 2011; 73:206-14.

10. Pagano N, Arosio M, Romeo F, et al. Balanced Propofol Sedation in Patients Undergoing EUS-FNA: A Pilot Study to Assess Feasibility and Safety. Diagn Ther Endosc. 2011; 2011:542159.

11. Jensen JT, Vilmann P, Horsted T, et al. Nurse-administered propofol sedation for endoscopy: a risk analysis during an implementation phase. Endoscopy. 2011; 43:716-22.

12. Heuss LT, Hanhart A, Dell-Kuster S, et al. Propofol sedation alone or in combination with pharyngeal lidocaine anesthesia for routine upper GI endoscopy: a randomized, double-blind, placebo-controlled, non-inferiority trial. Gastrointest Endosc. 201; 74:1207-14.

13. Martínez JF, Aparicio JR, Compañy L, et al. Safety of continuous propofol sedation for endoscopic procedures in elderly patients. Rev Esp Enferm Dig. 2011; 103:76-82.

14. Slagelse C, Vilmann P, Hornslet P, et al. Nurse-administered propofol sedation for gastrointestinal endoscopic procedures: first Nordic results from implementation of a structured training program. Scand J Gastroenterol. 2011; 46:1503-9.

15. Lee TH, Lee CK, Park SH, et al. Balanced propofol sedation versus propofol monosedation in therapeutic pancreaticobiliary endoscopic procedures. Dig Dis Sci. 2012; 57:2113-21.

16. Díez-Redondo P, Gil-Simón P, Alcaide-Suárez N, et al. Comparación entre la insuflación con aire ambiente o con dióxido de carbono durante la colonoscopia en pacientes sedados con propofol [Comparison between insufflation with air or carbon dioxide during the colonoscopy in sedated patients with propofol]. Rev Esp Enferm Dig. 2012; 104:411-7.

17. Friedrich K, Stremmel W, Sieg A. Endoscopist-administered propofol sedation is safe - a prospective evaluation of 10,000 patients in an outpatient practice. J Gastrointestin Liver Dis. 2012; 21:259-63.

18. Redondo-Cerezo E, Sánchez-Robaina A, Martínez Cara JG, et al. Gastroenterologist-guided sedation with propofol for endoscopic ultrasonography in average-risk and high-risk patients: a prospective series. Eur J Gastroenterol Hepatol. 2012; 24:506-12.

19. Levitzky BE, Lopez R, Dumot JA, et al. Moderate sedation for elective upper endoscopy with balanced propofol versus fentanyl and midazolam alone: a randomized clinical trial. Endoscopy. 2012; 44:13-20.

20. Lucendo AJ, Olveira A, Friginal-Ruiz AB, et al. Nonanesthesiologist-administered propofol sedation for colonoscopy is safe and effective: a prospective Spanish study over 1000 consecutive exams. Eur J Gastroenterol Hepatol. 2012; 24:787-92.

21. Molina-Infante J, Dueñas-Sadornil C, Mateos-Rodriguez JM, et al. Nonanesthesiologist-administered propofol versus midazolam and propofol, titrated to moderate sedation, for colonoscopy: a randomized controlled trial. Dig Dis Sci. 2012; 57:2385-93.

22. Bastaki M, Douzinas EE, Fotis TG, et al. A randomized double-blind trial of anesthesia provided for colonoscopy by university-degreed anesthesia nurses in Greece: safety and efficacy. Gastroenterol Nurs. 2013; 36:223-30.

23. González-Santiago JM, Martín-Noguerol E, Vinagre-Rodríguez G, et al. Intermittent boluses versus pump continuous infusion for endoscopist-directed propofol administration in colonoscopy. Rev Esp Enferm Dig. 2013; 105:378-84

24. Slagelse C, Vilmann P, Hornslet P, et al. The role of capnography in endoscopy patients undergoing nurse-administered propofol sedation: a randomized study. Scand J Gastroenterol. 2013; 48:1222-30.

25. Lucendo AJ, Arias Á, González-Castillo S, et al. Same-day bidirectional endoscopy with nonanesthesiologist administration of propofol: safety and cost-effectiveness compared with separated exams. Eur J Gastroenterol Hepatol. 2014; 26:301-8.

26. Yu YH, Han DS, Kim HS, et al. Efficacy of bispectral index monitoring during balanced propofol sedation for colonoscopy: a prospective, randomized controlled trial. Dig Dis Sci. 2013; 58:3576-83.

27. Kim YS, Kim MH, Jeong SU, et al. Comparison between Midazolam Used Alone and in Combination with Propofol for Sedation during Endoscopic Retrograde Cholangiopancreatography. Clin Endosc. 2014; 47:94-100.

28. Gotoda T, Kusano C, Nonaka M, et al. Non-anesthesiologist administrated propofol (NAAP) during endoscopic submucosal dissection for elderly patients with early gastric cancer. Gastric Cancer. 2014; 17:686-91.

29. Sieg A, Beck S, Scholl SG, et al. Safety analysis of endoscopist-directed propofol sedation: a prospective, national multicenter study of 24 441 patients in German outpatient practices. J Gastroenterol Hepatol. 2014; 29:517-23.

30. Khan HA, Umar M, Tul-Bushra H, et al. Safety of non-anaesthesiologist-administered propofol sedation in ERCP. Arab J Gastroenterol. 2014; 15:32-5.

31. Gurung RB, Purbe B, Malla B, et al. Safety profile and patient satisfaction of the routine use of propofol in gastrointestinal endoscopy. Kathmandu Univ Med J. 2014; 12:101-5.

32. de Paulo GA, Martins FP, Macedo EP, et al. Sedation in gastrointestinal endoscopy: a prospective study comparing nonanesthesiologist-administered propofol and monitored anesthesia care. Endosc Int Open. 2015;3: E7-E13.

33. Kawano S, Okada H, Iwamuro M, et al. An effective and safe sedation technique combining target-controlled infusion pump with propofol, intravenous pentazocine, and bispectral index monitoring for peroral double-balloon endoscopy. Digestion. 2015; 91:112-6.

34. Lee S, Han JH, Lee HS, et al. Efficacy and safety of a patient-positioning device (EZ-FIX) for endoscopic retrograde cholangiopancreatography. World J Gastroenterol. 2015; 21:5995-6000.

35. Ikeuchi N, Itoi T, Gotoda T, et al. Feasibility of non-anesthesiologist-administered propofol sedation for emergency endoscopic retrograde cholangiopancreatography. Gastroenterol Res Pract. 2015; 2015:685476.

36. Jensen JT, Møller A, Hornslet P, et al. Moderate and deep nurse-administered propofol sedation is safe. Dan Med J. 2015;62: A5049.

37. Nonaka M, Gotoda T, Kusano C, et al. F. Safety of gastroenterologist-guided sedation with propofol for upper gastrointestinal therapeutic endoscopy in elderly patients compared with younger patients. Gut Liver. 2015; 9:38-42.

38. Fanti L, Gemma M, Agostoni M, et al. Target Controlled Infusion for non-anaesthesiologist propofol sedation during gastrointestinal endoscopy: The first double blind randomized controlled trial. Dig Liver Dis. 2015; 47:566-71.

39. Okeke FC, Shaw S, Hunt KK, et al. Safety of Propofol Used as a Rescue Agent During Colonoscopy. J Clin Gastroenterol. 2016;50: e77-80.

40. Heo J, Jung MK, Lee HS, et al. Effects of bispectral index monitoring as an adjunct to nurse-administered propofol combined sedation during colonoscopy: a randomized clinical trial. Korean J Intern Med. 2016; 31:260-6.

41. Jensen JT, Hornslet P, Konge L, et al. High efficacy with deep nurse-administered propofol sedation for advanced gastroenterologic endoscopic procedures. Endosc Int Open. 2016;4: E107-11.

42. Klare P, Hartrampf B, Haller B, et al. Magnetic endoscope imaging for routine colonoscopy: impact on propofol dosage and patient safety - a randomized trial. Endoscopy. 2016; 48:916-22.

43. Ferreira AO, Torres J, Barjas E, et al. Non-anesthesiologist administration of propofol sedation for colonoscopy is safe in low risk patients: results of a noninferiority randomized controlled trial. Endoscopy. 2016; 48:747-53.

44. Seo SI, Ryu JY, Kang SS, et al. Safety of Target-Controlled Propofol Infusion by Gastroenterologists in Patients Undergoing Endoscopic Resection. Dig Dis Sci. 2016; 61:3199-3206.

45. Sathananthan D, Young E, Nind G, et al. Assessing the safety of physician-directed nurse-administered propofol sedation in low-risk patients undergoing endoscopy and colonoscopy. Endosc Int Open. 2017;5: E110-E115.

46. Han SJ, Lee TH, Park SH, et al. Efficacy of midazolam- versus propofol-based sedations by non-anesthesiologists during therapeutic endoscopic retrograde cholangiopancreatography in patients aged over 80 years. Dig Endosc. 2017; 29:369-376.

47. Kim MG, Park SW, Kim JH, et al. Etomidate versus propofol sedation for complex upper endoscopic procedures: a prospective double-blinded randomized controlled trial. Gastrointest Endosc. 2017; 86:452-461.

48. Behrens A, Kreuzmayr A, Manner H, et al. Acute sedation-associated complications in GI endoscopy (ProSed 2 Study): results from the prospective multicentre electronic registry of sedation-associated complications. Gut. 2019; 68:445-452.

49. López Muñoz C, Sánchez Yagüe A, Canca Sánchez JC, et al. Quality of sedation with propofol administered by non-anesthetists in a digestive endoscopy unit: the results of a one year experience. Rev Esp Enferm Dig. 2018; 110:231-236.

50. Patel J, Fang J, Taylor LJ, et al. Safety and efficacy of non-anesthesiologist administration of propofol sedation during esophagogastroduodenoscopy in the intensive care unit. Endosc Int Open. 2019;7: E625-E629.

51. Maestro Antolín S, Moreira Da Silva BA, Santos Santamarta F, et al. Severe cardiorespiratory complications derived from propofol sedation monitored by an endoscopist. Rev Esp Enferm Dig. 2018; 110:237-239.

52. Luzón Solanas L, Ollero Domenche L, Sierra Moros EM, et al. The safety of deep sedation with propofol controlled by the endoscopist in endoscopic retrograde cholangiopancreatography (ERCP): a prospective study in a tertiary hospital. Rev Esp Enferm Dig. 2018; 110:217-222.

53. López Rosés L, Álvarez B, González Ramírez A, et al. Viability of single balloon enteroscopy performed under endoscopist-directed sedation. Rev Esp Enferm Dig. 2018; 110:240-245.

54. Kim JH, Kim DH, Kim JH. Low-dose midazolam and propofol use for conscious sedation during diagnostic endoscopy. Kaohsiung J Med Sci. 2019; 35:160-167.

55. Takeuchi Y, Yamaguchi D, Yamaguchi N, et al. Propofol Sedation in the Endoscopy Room versus Operation Room during Endoscopic Submucosal Dissection for Gastric Tumors: A Propensity Score-Matching Analysis. Digestion. 2020; 101:450-457.

56. Lapidus A, Gralnek IM, Suissa A, et al. Safety and efficacy of endoscopist-directed balanced propofol sedation during endoscopic retrograde cholangiopancreatography. Ann Gastroenterol. 2019; 32:303-311.

57. Lee JG, Yoo KS, Byun YJ. Continuous infusion versus intermittent bolus injection of propofol during endoscopic retrograde cholangiopancreatography. Korean J Intern Med. 2020; 35:1338-1345.

58. Facciorusso A, Turco A, Barnabà C, et al. Efficacy and Safety of Non-Anesthesiologist Administration of Propofol Sedation in Endoscopic Ultrasound: A Propensity Score Analysis. Diagnostics (Basel). 2020; 10:791.

59. Riesco-López JM, Rizo-Pascual J, Díaz-Sánchez A, et al. Endoscopist-directed propofol is more efficient than anesthesiologist-administered propofol in patients at low-intermediate anesthetic risk. Eur J Gastroenterol Hepatol. 2020; 32:1440-1446.

60. Tiankanon K, Mekaroonkamol P, Pittayanon R, et al. Nurse Administered Propofol Sedation (NAPS) versus On-call Anesthesiologist Administered Propofol Sedation (OAPS) in Elective Colonoscopy. J Gastrointestin Liver Dis. 2020; 29:579-585.

61. Del Val Oliver B, González Valverde FM, Del Valle Ruiz SR. Safety of propofol sedation administered by an endoscopy team for outpatient colonoscopy. Rev Esp Enferm Dig. 2021; 113:385-386.

62. Manno M, Deiana S, Gabbani T, et al. Implementation of the European Society of Gastrointestinal Endoscopy (ESGE) and European Society of Gastroenterology and Endoscopy Nurses and Associates (ESGENA) sedation training course in a regular endoscopy unit. Endoscopy. 2021; 53:65-71.

63. Michael FA, Peveling-Oberhag J, Herrmann E, et al. Evaluation of the Integrated Pulmonary Index® during non-anesthesiologist sedation for percutaneous endoscopic gastrostomy. J Clin Monit Comput. 2021; 35:1085-1092.

64. Lee HS, Nagra N, La Selva D, et al. Nurse-Administered Propofol Continuous Infusion Sedation for Gastrointestinal Endoscopy in Patients Who Are Difficult to Sedate. Clin Gastroenterol Hepatol. 2021; 19:180-188.

65. Gururatsakul M, Lee R, Ponnuswamy SK, et al. Prospective audit of the safety of endoscopist-directed nurse-administered propofol sedation in an Australian referral hospital. J Gastroenterol Hepatol. 2021; 36:490-497.

66. Alam L, Khattak MA, Alam M. Safety of balanced propofol and midazolam in upper gastrointestinal endoscopy for sedation in cirrhotic patients. J Pak Med Assoc. 2021; 71:64-68.

67. Medina-Prado L, Martínez Sempere J, Bozhychko M, et al. Safety of endoscopist-administered deep sedation with propofol in ASA III patients. Rev Esp Enferm Dig. 2022; 114:468-473.

68. McKenzie P, Fang J, Davis J, et al. Safety of endoscopist-directed nurse-administered balanced propofol sedation in patients with severe systemic disease (ASA class III). Gastrointest Endosc. 2021; 94:124-130.

69. Steenholdt C, Jensen JT, Brynskov J, et al. Patient Satisfaction of Propofol Versus Midazolam and Fentanyl Sedation During Colonoscopy in Inflammatory Bowel Disease. Clin Gastroenterol Hepatol. 2022; 20:559-568

70. Fuentes-Valenzuela E, Pérez-Arenas E, de Benito Sanz M, et al. Prospective cohort study to evaluate premedication with simethicone and n-acetilcysteine for upper diagnostic gastrointestinal endoscopy. Rev Esp Enferm Dig. 2023; 115:10-15.

71. Behrens A, Ell Cet al. Safety of endoscopist-guided sedation in a low-risk collective. Z Gastroenterol. 2023; 61:1593-1602

72. Fatima H, Imperiale T. Safety Profile of Endoscopist-directed Balanced Propofol Sedation for Procedural Sedation: An Experience at a Hospital-based Endoscopy Unit. J Clin Gastroenterol. 2022;56: e209-e215.

73. Pozin IE, Zabida A, Nadler M, et al. Respiratory complications during recovery from gastrointestinal endoscopies performed by gastroenterologists under moderate sedation. Clin Endosc. 2023; 56:188-193.
