# Supplementary material for: The Incidence of Adverse Events in Adults Undergoing Procedural Sedation with Propofol Administered by Non-Anesthetists: A Systematic Review and Meta-Analysis
Source: Diagnostics (Basel). 2025 May 14;15(10):1234. doi: 10.3390/diagnostics15101234 (PMC12110594; doi:10.3390/diagnostics15101234)
Supplement: Supplementary file 1 [file diagnostics-15-01234-s001.zip › S3.pdf]

Appendix 3. Characteristics of the included studies

STUDY	YEAR	DESIGN	No. PATIENTS	PROCEDURES	MEDICATION USED	ADMNISTRATOR	WAY OF ADMINISTRATION	SUBGROUP ANALYSIS	AGE	BMI	ASA I/II	ASA III/IV
Akyuz [19]	2010	Retro	2918	EGDS Colon ERCP DBE	Mida Propofol	NuES	Bolus	NO	39.1±12.9*	NA	2.403	875
García-Suárez[6]	2010	Prosp	47	PEG	Propofol	NuES	Bolus	NO	82 (39-97)[$]	NA	0	47
Poincloux[7]	2011	RCT	45	Colon	Alfent Propofol	Endoscopist	Bolus	Endoscopist vs Anesthesist	56.2°	22.3[#]	45	0
Repici[8]	2011	Prosp	1593	Colon	Mida Propofol	Endoscopist	Bolus	NO	60 (22-75) [$]	NA	1593	0
Lee[9]	2011	RCT	102	EGDS ERCP	Mida Pethid Propofol	Nurse	Bolus	No propofol vs propofol	62±13*	23.3±3.4 *	91	11
Pagano[10]	2011	Prosp	112	EUS	Mida Propofol	Endoscopist	Bolus	NO	58 (25–86) [$]	NA	112	0
Jensen[11]	2011	Prosp	1764	EGDS Colon EUS ERCP DBE	Propofol	NuES	Bolus	NO	NA	<35	1.588	176
Heuss[12]	2011	RCT	294	EGDS	Propofol	Nurse	Bolus	Lydocaine spray vs placebo	61 ±18*	NA	146	116
Martínez[13]	2011	Prosp	1076	EGDS Colon EUS	Propofol	Endoscopist	TCI	>80 ys vs <80 ys	83.5(80-96)[$]; 57.5 (15-79) [$]	NA	905	171

Slagelse[14]	2011	Prosp	2527	EGDS Colon EUS ERCP DBE	Propofol	Nurse	Bolus	NO	Male 62 (15-95) $; Female (15-93) $	<35	2.301	236
Lee[15]	2012	RCT	206	EUS ERCP	Mida Fent Propofol	NuES	Bolus	BPS vs only propofol	65.0±15.3* 67.4±13.6*	23.2±3.4* 22.5±3.3*	164	42
Díez-Redondo [16]	2012	RCT	270	Colon	Mida Propofol	Endoscopist	Bolus	air vs CO2	56.7 (24-83) $ 56 (24-82) $	26#	NA	NA
Friedrich[17]	2012	Prosp	10000	EGDS Colon	Propofol	Nurse or Endoscopist	Bolus	NO	51.8°	NA	NA	NA
Redondo-Cerezo[18]	2012	Prosp	446	EUS	Propofol	NuES	Bolus	ASA I/II vs ASA III/IV	62±14*	NA	308	138
Levitzky[19]	2012	RCT	55	EGDS	Mida Fent Propofol	Endoscopist	Bolus	BPS vs no propofol	57 (51-62) $	26.0 (21.3-29.4)$	28	27
Lucendo[20]	2012	Prosp	1000	Colon	Propofol	NuES	Bolus	ASA I vs ASA II	57 (8-98) $	NA	1000	0
Molina-Infante [21]	2012	RCT	119	Colon	Mida Propofol	NuES	Bolus	Propofol vs BPS	56 (19–86) $	25.4±4.1*	104	15
Frieling[1]	2012	Prosp	191142	EGDS Colon	Mida Pethid Propofol	Nurse or Endoscopist	Bolus	NO	NA	NA	ND	ND
Bastaki[22]	2013	RCT	50	Colon	Propofol	Nurse	Bolus	Propofol vs no propofol	58.9 ±10.6*	26.15±4.03*	50	0
González-Santiago[23]	2013	RCT	192	Colon	Propofol	Nurse	Bolus/TCI	Bolus vs TCI	58 (22-89) $	25.6±4.2*	173	19
Slagelse[24]	2013	RCT	540	EGDS Colon	Propofol	Nurse	Bolus	Capnography vs no	59.4±15.9*	24.4±4.1*	502	38

								Capnography	59.1±16.5*	24.7±4.2*		
Lucendo[25]	2013	Prosp	1500	EGDS Colon	Propofol	NuES	Bolus	EGDS vs Colon vs Double examination	54(18–96)$	26#	1.266	234
Yu[26]	2013	RCT	115	Colon	Mida Propofol	NuES	Bolus	BIS vs no BIS	51.8±11.3*, 50.8±13.1*	23.8±3.0* vs 23.7±2.7*	108	7
Kim[27]	2014	Retro	50	ERCP	Mida Propofol	NuES	Bolus	Propofol vs no propofol	47.1±8.0*	NA	50	0
Gotoda[28]	2014	Retro	121	EGDS (ESD)	Pentaz Propofol	Endoscopist	TCI	<65 ys vs 65-75 ys vs >75 years	<65ys (n=32),<br>65-75ys (n=41),<br>>75ys (n= 48)	23.7±2.8*<br>22.9±3.3*<br>23.2±3.1*	118	3
Sieg[29]	2014	Prosp	24441	EGDS Colon	Mida Propofol	NuES	Bolus	NO	57.8°	<40	ND	ND
Khan[30]	2014	Prosp	156	ERCP	Propofol	Endoscopist	Bolus	NO	54.39±17.0*	24.65±21.62*	135	21
Gurung[31]	2014	Prosp	203	EGDS	Propofol	NuES	Bolus	NO	83%<60 ys	NA	NA	NA
Andrade de Paulo[32]	2014	Prosp	1000	EGDS Colon	Fent Propofol	Nurse	Bolus	NAAP vs Anesthesist	47.55 ±13.9*	23.97±4.09*	1000	0
Kawano[33]	2015	Prosp	34	Entero	Penthaz Propofol	Endoscopist	TCI	NO	56.2±15*	NA	ND	ND

Lee[34]	2015	RCT	105	ERCP	Mida Propofol	NuES	Bolus	EZ-FIX vs NO EZ-FIX	68.0 ± 15.4* 67.0 ± 14.0*	NA	80	25
Ikeuchi[35]	2015	Prosp	182	ERCP	Penthaz Propofol	Endoscopist	Bolus/TCI	mild vs severe cholangitis	75.4 ± 10.3*	22.7±3.7 *	76	106
Jensen[36]	2015	Retro	6840	EGDS Colon	Propofol	NuEs	Bolus	NO	59.8 ± 17.8*	NA	6.450	390
Ooi[4]	2015	Prosp	27989	EGDS Colon PEG	Mida Fent Propofol	NuES	Bolus	NO	57°	<35	NA	NA
Nonaka[37]	2015	Prosp	160	ERCP EGDS (ESD)	Pentaz Propofol	Endoscopist	Bolus	> 75 ys vs <75 ys	65.25±7.49* 80.75±4.29*	23.24±3.94* 22.98±3.27*	149	11
Fanti[38]	2015	RCT	70	EGDS Colon	Fent Propofol	Endoscopist	TCI	propofol vs no propofol	EGDS 47.8 ± 17.5* Colon 57.2 ± 13.8*	24.3±5* 25.4±6.4 *	70	0
Okeke[39]	2015	Retro	403	Colon	Mida Pethid Propofol	Endoscopist	Bolus	propofol vs no propofol	63.1± 0.5*	NA	NA	NA
Heo[40]	2016	RCT	280	Colon	Mida Pethidine Propofol	Nurse	Bolus	MOAAS vs BIS, Expert vs No Expert	54.3 ± 13.8*, 56.3 ± 10.3*, 56.2 ± 12.6*, 55.3 ± 12.1*	23.97±3.50* 23.54±2.84* 23.54±3.21* 24.02±3.85*	267	13
Jensen[41]	2016	Prosp	1899	ERCP EUS DBE	Propofol	Nurse	Bolus	NO	62.5±15.7*	<35	1.630	269

Klare[42]	2016	RCT	334	Colon	Propofol	Endoscopist	Bolus	MEI vs No MEI	54.2 ±17.4* 57.7 ±17.8*	24.9±5.0 * 25.0±5.5 *	287	47
Oliveira Ferreira[43]	2016	RCT	150	Colon	Propofol	NuES	Bolus	NAAP vs Anesthesis t	58.6 ±13.8*	NA	150	0
Seo[44]	2016	Retro	431	EGDS Colon (EMR/ESD)	Propofol	NuES	Bolus/TCI	Bolus vs TCI	57 (25–86) $ 63 (22–84) $	24.31(15.21–35.76)$ vs 24.72(18.25–33.56)$	393	38
Sathanant han[45]	2017	Prosp	981	EGDS Colon	Mida Propofol	NuES	Bolus	NO	53 (16-87) $	NA	965	16
Han[46]	2017	RCT	50	ERCP	Fent Propofol	Nurse	Bolus	propofol vs no propofol	83.96±3.75*	21.46±3.42*	43	7
Kim[47]	2017	RCT	64	EUS	Propofol	NuES	Bolus	Ethomidat e vs propofol	49.83±16.9*	23.51±4.31*	64	0
Behrens[48]	2018	Prosp	314.190 (84% with propofol)	EGDS Colon EUS ERCP Entero	Mida Propofol	Nurse or Endoscopist	ND	NO	65°	NA	248.774	65.416
López-Muñoz[49]	2018	Prosp	507	EGDS Colon ERCP EUS DBE	Propofol	NuES	ND	no propofol vs NAAP vs Anesthesis t	64°	NA	NA	NA
Sato[3]	2018	Prosp	150.211	EGDS Colon	Propofol	Nurse	Bolus	NO	59 (20–98) $; 65 (20–98) $	NA	150.211	0

Patel[50]	2018	Retro	161	EGDS	Fent Propofol	NuES	Bolus	NO	60.2 ± 14.0*	27.6±6.5 *	55	106
Ruiz-Curiel[2]	2018	Retro	70696	EGDS Colon ERCP EUS	Propofol	NuES	Bolus	NO	NA	NA	ND	ND
Maestro-Antolín[51]	2018	Retro	29524	EGDS Colon ERCP EUS	Propofol	Endoscopist	Bolus/TCI	NO	61.9±17.6* (egds) 63.9 ± 15.5* (Colon) 65.4 ± 14.4* (EUS) 75.3 ± 14.7* (ERCP)	NA	21.091	8.433
Luzón-Solanas [52]	2018	Prosp	661	ERCP	Propofol	Endoscopist	TCI	< 65 ys vs >65 ys	78.8(66.7-85.2) $	25.6(23.1-28.3)$	453	208
López-Rosés[53]	2018	Prosp	39	Entero	Mida Fent Propofol	NuES	TCI	NO	74 (18-89) $	NA	35	9
Kim[54]	2019	Retro	1000	EGDS Colon	Mida Pethid Propofol	Nurse or Endoscopist	Bolus	propofol vs no propofol	52.08±12.21* 50.10±9.78* 49.27±10.24* 50.02±9.36* 49.59±9.79*	NA	1000	0

Takeuchi[55]	2019	Retro	82	EGDS (ESD)	Pentaz Propofol	Endoscopist	TCI	NAAP vs Anesthesist	72.4±10.7*	NA	76	6
Lapidus[56]	2019	Retro	501	ERCP	Mida Fent Propofol	Endoscopist	Bolus	NAAP vs Anesthesist	64.1±18.9*	NA	445	56
Lee[57]	2020	RCT	232	ERCP	Mida Propofol	NuES	Bolus/TCI	Bolus vs TCI	65.8 ± 16.8* 68.9 ± 14.6*	23.1±3.4* 23.2±3.7*	ND	ND
Facciorusso[58]	2020	Prosp	305	EUS	Propofol	Nurse or Endoscopist	Bolus	NAAP vs Anesthesist	67 ± 3*	NA	252	53
Riesco-López[59]	2020	Prosp	1026	EGDS Colon EUS	Mida Fent Propofol	NuES	Bolus	NAAP vs Anesthesist	54.1±19.4*	NA	956	70
Tiankanon[60]	2020	Retro	189	Colon	Propofol	Nurse	TCI	NAAP vs Anesthesist	65.2 ± 12*	NA	189	0
Del Val Oliver[61]	2020	Retro	277	Colon	Propofol	NuES	ND	NO	NA	<40	277	0
Manno[62]	2020	Prosp	8471	EGDS Colon	Mida Fent Propofol	NuES	Bolus	propofol vs no propofol	60.5 ± 14.6*	25.7±4.3*	8.133	338
Michael [63]	2021	RCT	147	PEG	Mida Propofol	NuES	Bolus	Capnography vs no Capnography	NA	NA	NA	NA
Lee[64]	2021	Retro	1427	EGDS Colon	Fent Propofol	NuES	TCI	difficult to sedate vs not difficult to sedate	60.1± 12.5* 67.7 ± 11.3*	27.2±5.7* 29.1±15.2*	1.350	77

Gururatsaku[65]	2021	Prosp	24958	EGDS Colon	Mida Propofol	Nurse	Bolus	NO	(ASA 1) 48.85 ± 14.21* (ASA 2) 60.67 ± 12.97* (ASA 3) 66.60 ± 12.53* (ASA 4) 65.52 ± 15.85*	NA	23.221	1.737
Alam[66]	2021	Prosp	500	EGDS	Mida Propofol	Nurse or Endoscopist	Bolus	cirrhotics vs no cirrhotics	50.3 ± 18.1*	NA	330	170
Medina-Prado [67]	2021	Prosp	562	EGDS Colon EUS	Propofol	Endoscopist	TCI	ASA I/II vs ASA III	60.01±15.05*	30.8±0.8* 26.1±0.1*	482	80
McKenzie[68]	2021	Retro	24032	EGDS Colon	Fent Propofol	NuES	Bolus	ASA I/II vs ASA III	55.9 ±16.8* vs 49.3±17.6* EGDS ; 62.4±12.0* vs 56.5± 12.4* Colon	27.2(23.8-33.4)[$] 26.6(23.2-30.8)[$] EGDS; 30.8(26.3-36.2)[$] 27.0(23.7-30.9)[$] Colon	7.737	2.588
Steenholdt [69]	2022	RCT	63	Colon	Propofol	NuES	Bolus	propofol vs no propofol	41.9 ±13.3*	24.8±4.0*	63	0
Fuentes-Valenzuela [70]	2022	Prosp	205	EGDS	Propofol	Endoscopist	ND	NO	52.6°	25.6±4.9*	NA	NA

Behrens[71]	2022	RCT	28673	EGDS Colon	Mida Propofol	Nurse or Endoscopist	Bolus	Endoscopist vs Sedation Assistant	64#	NA	28.673	0
Fatima[72]	2022	Retro	1897	EGDS Colon	Mida Fent Propofol	NuES	Bolus	NO	54.98±11.40*	31.14±8.35*	1.562	335
Pozin[73]	2023	Retro	657	EGDS Colon EUS ERCP DBE	Mida Fent Propofol	NuES	Bolus	NO	63.0±15.3*	26.8±5.2 *	386	271

Prosp (Prospective observational study), Retro (Retrospective), RCT (Randomized Controlled Trial), EGDS (Esophagogastroduodenoscopy), Colon (Colonoscopy), EUS (Endoscopic Ultrasound), ERCP (Endoscopic Retrograde ColangioPancreatography), PEG (Percutaneous Endoscopic Gastrostomy), Entero (Enteroscopy), DBE (Double Balloon Enteroscopy), ESD (Endoscopic submucosal Dissection), EMR (Endoscopic Mucosal Resection), Mida (Midazolam), Fent (Fentanyl), Pethid (Pethidine), Penthaz (Penthazocine), NuES (Nurse under Endoscopist Supervision), TCI (Target Controlled Infusion). Results expressed as mean (°), mean ± standard deviation (*), median (#), median min-max ($), BPS (Balances Propofol Sedation), BIS (Bispectral Index), NAAP (Non-Aneshtesiologist Administered Propofol), MOAAS (Modified Observer's Assessment of Alertness/Sedation Scale), MEI (Magnetic Endoscopic Imaging)
