# Supplementary material for: The Incidence of Adverse Events in Adults Undergoing Procedural Sedation with Propofol Administered by Non-Anesthetists: A Systematic Review and Meta-Analysis
Source: Diagnostics (Basel). 2025 May 14;15(10):1234. doi: 10.3390/diagnostics15101234 (PMC12110594; doi:10.3390/diagnostics15101234)
Supplement: Supplementary file 1 [file diagnostics-15-01234-s001.zip › S4.pdf]

Appendix 4. Characteristics of outcomes of included studies

STUDY	YEAR	HYPOTHENSION	HEART RATE	HYPOXIA	MAJOR ADVERSE EVENTS
Akyuz [19]	2010	Sp <90 mmHg	Hr<50 bpm	ND	ETI
García-Suárez[6]	2010	Sp <90 mmHg	Hr<50 bpm	SO_2<90%	ND
Poincloux[7]	2011	Map <85% of baseline	ND	SO_2<95% >30 sec	ETI, Death
Repici[8]	2011	Sp <90 mmHg	Hr<60 bpm	SO_2<90% >30 sec	Assisted ventilation, Medical treatment
Lee[9]	2011	Sp <90 mmHg	Hr<50 bpm	SO_2<90%	Permanent Disability, Death
Pagano[10]	2011	Sp <90 mmHg	Hr<50 bpm	SO_2<85% >20 sec	ND
Jensen[11]	2011	Sp Delta >20 mmHg	ND	SO_2<92%	Anaesthetist call
Heuss[12]	2011	ND	ND	SO_2<85%	ND
Martínez[13]	2011	Sp <90 mmHg	Hr<40 bpm >10 sec	SO_2<90% (Mild), SO_2<85% >10 sec (Severe)	ND
Slagelse[14]	2011	Sp Delta >30 mmHg	Hr<40 bpm	SO_2 92-90%, $SO_2$90-88%, SO_2<88%	nd
Lee[15]	2012	Sp <90 mmHg	Hr<50 bpm	SO_2<90%	Permanent Disability, Death
Díez-Redondo [16]	2012	ND	ND	SO_2<90%	ND
Friedrich[17]	2012	Sp <80 mmHg	Hr<40 bpm	SO_2<90% >30 sec	ND
Redondo-Cerezo[18]	2012	Sp <90 mmHg	Hr<50 bpm	SO_2<90%	ETI, Permanent disability, recovery, death
Levitzky[19]	2012	Sp Delta >25%	Hr<25%	SO_2<90%	ND
Lucendo[20]	2012	Sp <90 mmHg or Delta >20%	Hr<50 bpm	SO_2<90% >10 sec	Assisted ventilation, medical treatment
Molina-Infante [21]	2012	Sp <80mmHg	Hr<40 bpm	SO_2<85% >30 sec	ETI, Anaesthetist call, Death
Frieling[1]	2012	ND	ND	ND	Assisted ventilation, ETI, death
Bastaki[22]	2013	Sp <90 mmHg OR Dp <60 mmHg	Hr<60 bpm	SO_2<90%	ETI
González-Santiago[23]	2013	Sp <90 mmHg	Hr<50 bpm	SO_2<85%> 30 sec	Anaesthetist call, ETI

Slagelse[24]	2013	ND	ND	SO_2<88%	ND
Lucendo[25]	2013	Sp <90 mmHg	Hr<50 bpm	SO_2<90%>10 sec	Assisted ventilation, medical treatment
Yu[26]	2013	Sp <90 mmHg	Hr<50 bpm	SO_2<90%	ND
Kim[27]	2014	Sp Delta>20 mmHg	Hr Delta>50%	SO_2<90% >10 sec	ND
Gotoda[28]	2014	Sp <80 mmHg	Hr<40 bpm	SO_2<90%>10 sec	ND
Sieg[29]	2014	Sp <90 mmHg >30 sec	Hr<50 bpm	SO_2<90% >2 minutes or SO_2<90% with medical intervention	Assisted ventilation, ETI
Khan[30]	2014	ND	Hr<50 bpm	SO_2<90%	ETI, Death
Gurung[31]	2014	ND	ND	SO_2 90-95%, SO_2 86-89%, SO_2<85%	ND
Andrade de Paulo[32]	2014	Map delta>20%	ND	SO_2<85%	ND
Kawano[33]	2015	Sp <80 mmHg	Hr<50 bpm	SO_2<90% >10 sec	ETI, Permanent disability, recovery, death
Lee[34]	2015	Sp delta>20 mmHg	Hr Delta>20%	SO_2<90% >30 sec	Death
Ikeuchi[35]	2015	Sp <80 mmHg	Hr<50 bpm	SO_2<90%	ND
Jensen[36]	2015	Sp <80 mmHg or MAP Delta>30%	ND	SO_2<92%	ETI
Ooi[4]	2015	Sp <90 mmHg	Hr<40 bpm	SO_2<90%	ETI, Death
Nonaka[37]	2015	Sp <80 mmHg	Hr<50 bpm	SO_2<90% >10 sec	ND
Fanti[38]	2015	Map<60 mm Hg	Hr<50 bpm	SO_2<90%	ND
Okeke[39]	2015	Sp <90 mmHg OR Dp <60 mmHg	ND	SO_2<90%	Hypotension not responsive to liquid infusion, assisted ventilation, ETI
Heo[40]	2016	Sp <90 mmHg	Hr<50 bpm	SO_2<80% >15 sec	ND
Jensen[41]	2016	Sp Delta>50 mmHg or Map Delta>30%	ND	SO_2<92%	Anaesthetist call, ETI
Klare[42]	2016	Sp <90 mmHg	Hr<50 bpm	SO_2<90%	ND
Oliveira Ferreira[43]	2016	Sp Delta>25%	Hr Delta > 25%	SO_2<90% <60 sec	SO_2<90% <60 sec, SO_2<75%, cardiac, death
Seo[44]	2016	Sp <80 mmHg	ND	SO_2<90%	SO_2<85% with assisted ventilation, hypotension with medical treatment,

					ETI, resuscitation, permanent disability, death
Sathananthan[45]	2017	Sp <100 mmHg or Delta> 20 mmHg	Hr<50 bpm	SO_2<90% >10 sec	Anaesthetist call, ETI, Permanent disability, recovery, death
Han[46]	2017	Sp <90 mmHg	Hr<50 bpm	SO_2<90%	Permanent disability, death
Kim[47]	2017	Sp <90 mmHg	Hr<50 bpm	SO_2<90%	ETI, Permanent disability, death
Behrens[48]	2018	Sp Delta>25%	Hr Delta> 20%	SO_2<90% >10 sec	ETI, resuscitation, death
López-Muñoz[49]	2018	Sp <90 mmHg or Delta>20%	ND	SO_2<85%	ETI, death
Sato[3]	2018	ND	Hr<50 bpm	SO_2<90% >20 sec	Assisted ventilation, ETI
Patel[50]	2018	Sp <90 mmHg	Hr<60 bpm	SO_2<90%	Assisted ventilation, ETI
Ruiz-Curiel[2]	2018	ND	ND	ND	ETI
Maestro-Antolín[51]	2018	ND	ND	SO_2<88%	ETI
Luzón-Solanas [52]	2018	Sp <90 mmHg or Delta >25 mmHg	Hr<50 bpm	SO_2<90%	Death
López-Rosés[53]	2018	Sp <90 mmHg	Hr<50 bpm	SO_2<90%, SO_2 80-90%, SO_2<80%	ND
Kim[54]	2019	ND	Hr Delta> 20%	SO_2<90% >20 sec	ND
Takeuchi[55]	2019	Sp <80 mmHg	Hr<60 bpm	SO_2<90%	Death
Lapidus[56]	2019	ND	ND	ND	Assisted ventilation, ETI, recovery
Lee[57]	2020	Sp <90 mmHg	Hr<60 bpm	SO_2<90%	ETI
Facciorusso[58]	2020	Sp Delta>25%	Hr Delta> 20%	SO_2<90% >5 sec	ETI, permanent disability, recovery, death
Riesco-López[59]	2020	Sp <90 mmHg	Hr<40 bpm	SO_2<90%	Anaesthetist call, ETI, death
Tiankanon[60]	2020	Sp <90 mmHg or Delta>50 mmHg or Map Delta >25%	Hr Delta> 20%	$SO_2$75–90% > 20-60 sec	$SO_2$75–90% > 60 sec, SO_2 <75%, cardiac arrest
Del Val Oliver[61]	2020	Sp <90 mmHg	Hr<50 bpm	SO_2<90%	ND

Manno[62]	2020	Sp Delta>25%	Hr<50 bpm	$SO_2$75-90 % <60 sec with no invasive interventions; with invasive interventions (moderate)	SO_2<90%0>60 sec, SO_2<75%, cardiac arrest, shock, permanent disability, death
Michael [63]	2021	Sp <100 mmHg	Hr<50 bpm	SO_2<90% >15 sec, SO_2<85% (severe)	ND
Lee[64]	2021	Sp <90 mmHg	ND	SO_2<90%	ND
Gururatsaku[65]	2021	Sp <90 mmHg or Delta>20 mmHg	Hr<50 bpm	SO_2<90% >30 sec	Anaesthetist call, cardiac arrest, death
Alam[66]	2021	Sp <90 mmHg	Hr<60 bpm	SO_2<90%	SO_2<90% >30 sec
Medina-Prado [67]	2021	Sp <90 mmHg	Hr<50 bpm	SO_2<90%, SO_2<80% (severe)	ETI
McKenzie[68]	2021	Sp <90 mmHg	Hr<50 bpm	SO_2<90%	ETI, death
Steenholdt [69]	2022	Delta Map> 30%	ND	SO_2<92%	ND
Fuentes-Valenzuela[70]	2022	ND	ND	SO_2<90%	ND
Behrens[71]	2022	Delta Map>25%	Hr Delta>20%	SO_2<90% >10 sec	ETI, resuscitation, death
Fatima[72]	2022	Sp <90 mmHg or Delta>50 mmHg	Hr<40 bpm	SO_2<90%	ETI, permanent disability, recovery, death
Pozin[73]	2023	ND	ND	SO_2<90% <60 sec, SO_2<90% >60 sec, SO_2<75%	ND

Sp (Systolic pressure), Dp (Diastolic pressure), Map (Mean Arterial Pressure), Hr (Heart rate), ND (not defined), ETI (EndoTracheal Intubation), SO_2 (saturation oxygen)
