# Supplementary material for: The Incidence of Adverse Events in Adults Undergoing Procedural Sedation with Propofol Administered by Non-Anesthetists: A Systematic Review and Meta-Analysis
Source: Diagnostics (Basel). 2025 May 14;15(10):1234. doi: 10.3390/diagnostics15101234 (PMC12110594; doi:10.3390/diagnostics15101234)
Supplement: Supplementary file 1 [file diagnostics-15-01234-s001.zip › S5.pdf]

Appendix 5. Cochrane Collaboration's tool for assessment of risk of bias in randomized controlled trials

Study	Adequate Sequence Generation	Allocation concealment	Blinding of participants, personnel and outcome assessors	Incomplete Outcome Data addressed	Free of selective outcome reporting	Free of other bias/ Bias
Poincloux 2011[7]	Yes	Unclear	Unclear	Yes	Yes	Yes
Lee 2011[9]	Yes	Yes	No	Yes	Yes	Yes
Heuss 2011[12]	Yes	Yes	Yes	Yes	No	No/level of sedation and extent not measured
Lee 2012[15]	Yes	Yes	Yes	Yes	No	No/level of sedation not measured
Diez-Redondo 2012[16]	Yes	Yes	Yes	Yes	Yes	No
Levitzky 2012[19]	Yes	Unclear	No	Yes	No	No/ VAS scale not completely attendable
Molina Infante 2012[21]	Yes	Yes	Yes	Yes	Yes	No
Bastaki 2012[22]	Yes	Yes	Unclear	Yes	No	No/onset time of sedation and recovery not comparable
Gonzalez-Santiago 2013[23]	Yes	Yes	Yes	Yes	No	No/differences between groups, such as propofol doses or depth of sedation
Slagelse 2013[24]	Yes	Yes	No	Yes	Yes	Yes
Yu 2013[26]	Yes	Yes	No	Yes	Yes	Yes
Lee 2015[34]	Unclear	Unclear	No	Yes	Yes	Yes
Fanti 2015[38]	Yes	Yes	Yes	Yes	Yes	Yes
Heo 2016[40]	Yes	Yes	No	Yes	Yes	Yes
Klare 2016[42]	Yes	Yes	No	Yes	Bo	No/ difference in comorbidities
Oliveira-Ferreira 2016[43]	Yes	Yes	No	Yes	No	No/ disparity in number of endoscopist in each group
Han 2017[46]	Yes	Yes	No/not blinded to repeated injection	Yes	Yes	Yes
Kim 2017[47]	Yes	Yes	incomplete blindness, attributed to the predominant adverse events of each medication	Yes	Yes	Yes
Lee 2020[57]	Yes	Yes	Yes	no objective	Yes	No/direct

				indicators to assess quality of sedation		comparison of the total dose of propofol between the two groups may be inappropriate because midazolam may have had a propofol-sparing effect
Michael 2021[63]	Yes	Yes	single blind	Yes	Yes	Yes
Steenholdt 2022[69]	Yes	Yes	unblinded	Yes	Yes	Yes
Behrens 2022[71]	Yes	Yes	Yes	No/reporting system of delayed adverse events not well structered	Yes	No/ small study size
