# Supplementary material for: The Incidence of Adverse Events in Adults Undergoing Procedural Sedation with Propofol Administered by Non-Anesthetists: A Systematic Review and Meta-Analysis
Source: Diagnostics (Basel). 2025 May 14;15(10):1234. doi: 10.3390/diagnostics15101234 (PMC12110594; doi:10.3390/diagnostics15101234)
Supplement: Supplementary file 1 [file diagnostics-15-01234-s001.zip › S6.pdf]

Appendix 6. Risk of bias assessment for cohort studies using the Newcastle Ottawa scale

Article	Selection	Comparability	Exposure/Outcome
Akyuz 2010[5]	3	NA	2
Garcia Suarez 2010[6]	3	NA	3
Repici 2011[8]	4	NA	2
Pagano 2011[10]	4	NA	2
Jensen 2011[11]	3	NA	2
Martinez 2011[13]	4	1	2
Slagelse 2011[14]	3	1	3
Redondo Cerezo 2012[18]	4	NA	2
Lucendo 2012[20]	4	1	3
Friedrich 2012[17]	4	NA	2
Frieling 2013[8]	2	NA	1
Lucendo 2012[20]	3	1	3
Kawano 2015[33]	4	NA	2
Kim 2014[27]	4	1	2
Khan 2014[30]	3	NA	2
Gotoda 2014[28]	3	NA	2
Sieg 2014[29]	4	NA	3
Andrade de Paulo 2014[32]	3	1	3
Gurung 2014[31]	3	NA	1
Ikeuchi 2015[35]	4	NA	2
Ooi 2015[4]	4	NA	3
Jensen 2015[36]	4	NA	2
Okeke 2015[39]	2	1	2
Nonaka 2015[37]	3	1	2
Seo 2016[44]	3	1	3
Jensen 2016[41]	4	1	2
Sathananthan 2017[45]	4	NA	2
Patel 2018[50]	4	NA	2

Behrens 2018[48]	4	NA	3
Lopez-Munoz 2018[49]	3	1	2
Kim 2019[54]	4	1	3
Sato 2018[3]	3	NA	1
Maestro-Antolin 2018[51]	2	NA	1
Luzon-Solanas 2018[52]	3	1	3
Ruiz-Curiel 2018[2]	2	NA	1
Lopez-Roses 2018[53]	3	NA	2
Takeuchi 2019[55]	3	1	3
Lapidus 2019[56]	2	1	1
Manno 2020[62]	3	1	3
Facciorusso 2020[58]	4	1	3
Riesco-Lopez 2020[59]	4	1	2
Tiankanon 2020[60]	3	1	3
Del Val Oliver 2020[61]	3	NA	1
Medina Prado 2021[67]	4	1	2
McKenzie 2021[68]	4	NA	3
Lee 2021[64]	3	1	2
Alam 2021[66]	3	1	1
Fatima 2021[72]	4	NA	3
Gururatsakul 2021[65]	3	NA	3
Fuentes-Valenzuela 2022[70]	3	NA	1
Behrens 2022[71]	4	1	3
Pozin 2023[73]	3	NA	1
