# Supplementary material for: The Incidence of Adverse Events in Adults Undergoing Procedural Sedation with Propofol Administered by Non-Anesthetists: A Systematic Review and Meta-Analysis
Source: Diagnostics (Basel). 2025 May 14;15(10):1234. doi: 10.3390/diagnostics15101234 (PMC12110594; doi:10.3390/diagnostics15101234)
Supplement: Supplementary file 1 [file diagnostics-15-01234-s001.zip › S7.pdf]

Appendix 7. Incidence of hypoxia among different saturation oxygen (SO2) cut-off definition (per 1,000 Procedural Sedations)

	**95%< SO2 < 91%**	**90%< SO2 < 86%**	**81%< SO2 < 85%**	**SO2≤80%**
Events/Total	424/13.341	4.250/855.460	576/38.788	66/34.230
Estimate per 1.000 (‰)	37	37	48	6
95%CI	19-61	31-43	25-76	1-13
I^2(%)	96	99	98	94

Results are presented as number of events over the total of patients (only studies that reported the events), estimate on 1.000 patients, 95% Confidence interval and heterogeneity index (I^2)
