# Supplementary material for: The Incidence of Adverse Events in Adults Undergoing Procedural Sedation with Propofol Administered by Non-Anesthetists: A Systematic Review and Meta-Analysis
Source: Diagnostics (Basel). 2025 May 14;15(10):1234. doi: 10.3390/diagnostics15101234 (PMC12110594; doi:10.3390/diagnostics15101234)
Supplement: Supplementary file 1 [file diagnostics-15-01234-s001.zip › S8.pdf]

Appendix 8. Forest plot of estimation of incidence of hypoxia with respect to classes of oxygen saturation and representation of weight of single study

Study or Subgroup	Events	Total	Weight	IV, Random, 95% CI
**Subgroup = 95%<S02<91%**				
Poincloux/Alfent Prop 2011	3	45	1.0%	0.07 [0.01; 0.18]
Jensen/Propofol 2011	77	1764	1.2%	0.04 [0.03; 0.05]
Slagelse/Propofol 2011	17	2527	1.2%	0.01 [0.00; 0.01]
Gurung /Propofol 2014	32	203	1.1%	0.16 [0.11; 0.22]
Jensen/Propofol 2015	214	6840	1.2%	0.03 [0.03; 0.04]
Jensen/Propofol 2016	81	1899	1.2%	0.04 [0.03; 0.05]
Steenholdt /Propofol 2022	0	63	1.0%	0.00 [0.00; 0.06]
Total (95% CI)		13341	8.0%	0.04 [0.01; 0.08]
Heterogeneity: $Tau^2 = 0.0119$; $Chi^2 = 152.97$, df = 6 ($P < 0.01$); $I^2 = 96\%$				
**Subgroup = 90%<S02<86%**				
García-Suárez/Propofol 2010	8	47	1.0%	0.17 [0.08; 0.31]
Repici/Midaz Prop 2011	8	1593	1.2%	0.01 [0.00; 0.01]
Lee/Midaz Pethid Prop 2011	6	102	1.1%	0.06 [0.02; 0.12]
Martínez /Propofol 2011	154	1076	1.2%	0.14 [0.12; 0.17]
Slagelse/Propofol 2011	119	2527	1.2%	0.05 [0.04; 0.06]
Lee/Midaz Fent Prop 2012	13	206	1.2%	0.06 [0.03; 0.11]
Díez-Redondo /Midaz Prop 2012	57	270	1.2%	0.21 [0.16; 0.26]
Friedrich/Propofol 2012	42	10000	1.2%	0.00 [0.00; 0.01]
Redondo-Cerezo/Propofol 2012	36	446	1.2%	0.08 [0.06; 0.11]
Levitzky/Midaz Fent Prop 2012	7	55	1.0%	0.13 [0.05; 0.24]
Lucendo/Propofol 2012	24	1000	1.2%	0.02 [0.02; 0.04]
Frieling/Midaz Pethid Prop 2012	82	191142	1.2%	0.00 [0.00; 0.00]
Bastaki/Propofol 2013	1	50	1.0%	0.02 [0.00; 0.11]
González-Santiago/Propofol 2013	11	192	1.1%	0.06 [0.03; 0.10]
Slagelse/Propofol 2013	45	540	1.2%	0.08 [0.06; 0.11]
Lucendo/Propofol 2013	60	1500	1.2%	0.04 [0.03; 0.05]
Yu/Midaz Prop 2013	12	115	1.1%	0.10 [0.06; 0.18]
Kim/Midaz Prop 2014	1	50	1.0%	0.02 [0.00; 0.11]
Gotoda/Pentaz Prop 2014	12	121	1.1%	0.10 [0.05; 0.17]
Sieg/Midaz Prop 2014	93	24441	1.2%	0.00 [0.00; 0.00]
Khan/Propofol 2014	1	156	1.1%	0.01 [0.00; 0.04]
Gurung /Propofol 2014	8	203	1.1%	0.04 [0.02; 0.08]
Kawano/Pentaz Prop 2015	1	34	0.9%	0.03 [0.00; 0.15]
Lee/Midaz Prop 2015	10	105	1.1%	0.10 [0.05; 0.17]
Ikeuchi/Pentaz Prop 2015	31	182	1.1%	0.17 [0.12; 0.23]
Ooi/Midaz Fent Prop 2015	12	27989	1.2%	0.00 [0.00; 0.00]
Nonaka/Pentaz Prop 2015	26	160	1.1%	0.16 [0.11; 0.23]
Fanti/Fent Prop 2015	0	70	1.0%	0.00 [0.00; 0.05]
Okeke/Midaz Pethid Prop 2015	0	403	1.2%	0.00 [0.00; 0.01]
Klare/Propofol 2016	27	334	1.2%	0.08 [0.05; 0.12]
Oliveira Ferreira/Propofol 2016	14	150	1.1%	0.09 [0.05; 0.15]
Seo/Propofol 2016	7	431	1.2%	0.02 [0.01; 0.03]
Sathananthan/Midaz Prop 2017	1	981	1.2%	0.00 [0.00; 0.01]
Han/Fent Prop 2017	21	50	1.0%	0.42 [0.28; 0.57]
Behrens/Midaz Prop 2018	338	314190	1.2%	0.00 [0.00; 0.00]
Sato/Propofol 2018	1950	150211	1.2%	0.01 [0.01; 0.01]
Patel/Fent Prop 2018	12	161	1.1%	0.07 [0.04; 0.13]
Maestro-Antolín/Propofol 2018	39	29524	1.2%	0.00 [0.00; 0.00]
Luzón-Solanas /Propofol 2018	38	661	1.2%	0.06 [0.04; 0.08]
López-Rosés,/Midaz Fent Prop 2018	2	39	0.9%	0.05 [0.01; 0.17]
Kim/Midaz Pethid Prop 2019	49	1000	1.2%	0.05 [0.04; 0.06]
Takeuchi /Pentaz Prop 2019	15	82	1.1%	0.18 [0.11; 0.28]
Lee/Midaz Prop 2020	10	232	1.2%	0.04 [0.02; 0.08]
Facciorusso/Propofol 2020	13	305	1.2%	0.04 [0.02; 0.07]
Riesco-López/Midaz Fent Prop 2020	39	1026	1.2%	0.04 [0.03; 0.05]
Tiankanon /Propofol 2020	4	189	1.1%	0.02 [0.01; 0.05]
Del Val Olive/Propofol 2020	4	277	1.2%	0.01 [0.00; 0.04]
Manno/Midaz Fent Prop 2020	7	8471	1.2%	0.00 [0.00; 0.00]
Michael /Midaz Prop 2021	43	147	1.1%	0.29 [0.22; 0.37]
Lee/Fent Prop 2021	14	1427	1.2%	0.01 [0.01; 0.02]
Gururatsaku/Midaz Prop 2021	41	24958	1.2%	0.00 [0.00; 0.00]
Alam/Midaz Prop 2021	4	500	1.2%	0.01 [0.00; 0.02]
Medina-Prado /Propofol 2021	47	562	1.2%	0.08 [0.06; 0.11]
McKenzie /Fent Prop 2021	248	24032	1.2%	0.01 [0.01; 0.01]
Fuentes-Valenzuela/Propofol 2022	4	205	1.1%	0.02 [0.01; 0.05]
Behrens/Midaz Prop 2022	241	28673	1.2%	0.01 [0.01; 0.01]
Fatima/Midaz Fent Prop 2022	65	1897	1.2%	0.03 [0.03; 0.04]
Pozin/Midaz Fent Prop 2023	161	657	1.2%	0.25 [0.21; 0.28]
Total (95% CI)		856147	66.6%	0.04 [0.03; 0.06]
Heterogeneity: $Tau^2 = 0.0213$; $Chi^2 = 7656.96$, df = 57 ($P = 0$); $I^2 = 99\%$				
**Subgroup = 85%<S02<81%**				
Pagano/Midaz Prop 2011	5	112	1.1%	0.04 [0.01; 0.10]
Heuss/Propofol 2011	10	294	1.2%	0.03 [0.02; 0.06]
Martínez /Propofol 2011	43	1076	1.2%	0.04 [0.03; 0.05]
Slagelse/Propofol 2011	51	2527	1.2%	0.02 [0.02; 0.03]
Molina-Infante /Midaz Prop 2012	10	119	1.1%	0.08 [0.04; 0.15]
Gurung /Propofol 2014	1	203	1.1%	0.00 [0.00; 0.03]
Andrade de Paulo/Fent Prop 2014	128	1000	1.2%	0.13 [0.11; 0.15]
López-Muñoz/Propofol 2018	12	507	1.2%	0.02 [0.01; 0.04]
López-Rosés,/Midaz Fent Prop 2018	2	39	0.9%	0.05 [0.01; 0.17]
Tiankanon /Propofol 2020	4	189	1.1%	0.02 [0.01; 0.05]
Manno/Midaz Fent Prop 2020	3	8471	1.2%	0.00 [0.00; 0.00]
Michael /Midaz Prop 2021	31	147	1.1%	0.21 [0.15; 0.29]
Medina-Prado/Propofol 2021	26	562	1.2%	0.05 [0.03; 0.07]
McKenzie /Fent Prop 2021	248	24032	1.2%	0.01 [0.01; 0.01]
Pozin/Midaz Fent Prop 2023	161	657	1.2%	0.25 [0.21; 0.28]
Total (95% CI)		39935	17.4%	0.05 [0.02; 0.08]
Heterogeneity: $Tau^2 = 0.0191$; $Chi^2 = 1175.87$, df = 14 ($P < 0.01$); $I^2 = 99\%$				
**Subgroup = S02<80%**				
Heo/Midaz Pethid Prop 2016	0	280	1.2%	0.00 [0.00; 0.01]
López-Rosés/Midaz Fent Prop 2018	2	39	0.9%	0.05 [0.01; 0.17]
Tiankanon /Propofol 2020	4	189	1.1%	0.02 [0.01; 0.05]
Manno/Midaz Fent Prop 2020	3	8471	1.2%	0.00 [0.00; 0.00]
Medina-Prado /Propofol 2021	26	562	1.2%	0.05 [0.03; 0.07]
McKenzie /Fent Prop 2021	27	24032	1.2%	0.00 [0.00; 0.00]
Pozin/Midaz Fent Prop 2023	4	657	1.2%	0.01 [0.00; 0.02]
Total (95% CI)		34230	8.1%	0.01 [0.00; 0.03]
Heterogeneity: $Tau^2 = 0.0068$; $Chi^2 = 107.57$, df = 6 ($P < 0.01$); $I^2 = 94\%$				
**Total (95% CI)**		**943653**	**100.0%**	**0.04 [0.03; 0.05]**
**Prediction interval**				**[0.00; 0.22]**

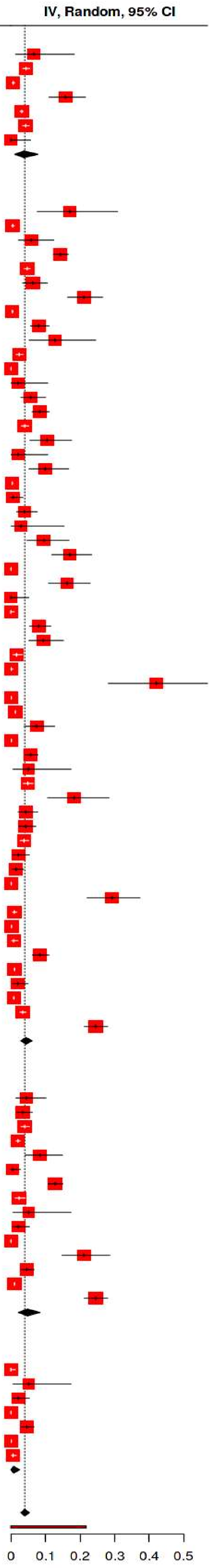

Heterogeneity: $Tau^2 = 0.0194$; $Chi^2 = 10322.88$, df = 86 ($P = 0$); $I^2 = 99\%$
Test for subgroup differences: $Chi^2 = 9.70$, df = 3 ($P = 0.02$)
