# Supplementary material for: The Incidence of Adverse Events in Adults Undergoing Procedural Sedation with Propofol Administered by Non-Anesthetists: A Systematic Review and Meta-Analysis
Source: Diagnostics (Basel). 2025 May 14;15(10):1234. doi: 10.3390/diagnostics15101234 (PMC12110594; doi:10.3390/diagnostics15101234)
Supplement: Supplementary file 1 [file diagnostics-15-01234-s001.zip › S9.pdf]

Appendix 9. Incidence of adverse events among different sedation regimen (Estimate per 1,000 Procedural Sedations)

Adverse Events	Midazolam/ Propofol	Midazolam/ Opioid/ Propofol	Opioid/ Propofol	Balanced propofol sedation	Propofol	p-value
Hypoxia						
Events/Total	905/396.486	450/233.267	538/27.364	1.893/657.117	3.208/282.713	0,45
Estimate per 1.000 (‰)	29	26	87	37	42	
95%CI	20-39	15-41	43-143	30-43	32-54	
I^2(%)	98	99	97	98	99	
Hypotension						
Events/Total	1.012/398.987	300/41.125	2.550/27.364	3.862/467.476	1.467/102.030	0,48
Estimate per 1.000 (‰)	35	12	96	42	33	
95%CI	20-52	0,5-35	43-17	28-60	14-40	
I^2(%)	99	99	98	99	99	
Bradycardia						
Events/Total	170/398.987	20/41.722	1093/26.364	1.283/467.073	303/174.861	0,36
Estimate per 1.000 (‰)	4	0,1	22	8	13	
95%CI	2-7	0,5-1,4	0,5-47	4-14	6-22	
I^2(%)	97	86	94	99	97	
Major						
Events/Total	51/399.257	15/233.768	10/26.364	76/659.389	33/282.173	0,20
Estimate per 1.000 (‰)	0,1	0,1	1	0,1	0,1	
95%CI	0,05-0,2	0,001-0,12	0,5-5	0,05-0,2	0,05-0,3	
I^2(%)	0	10	79	52	78	

Results are presented as number of events over the total of patients (only studies that reported the events), estimate on 1.000 patients, 95% Confidence interval and heterogeneity index (I^2); p-value calculated between Balanced propofol sedation vs Propofol only
