# Supplementary material for: The Incidence of Adverse Events in Adults Undergoing Procedural Sedation with Propofol Administered by Non-Anesthetists: A Systematic Review and Meta-Analysis
Source: Diagnostics (Basel). 2025 May 14;15(10):1234. doi: 10.3390/diagnostics15101234 (PMC12110594; doi:10.3390/diagnostics15101234)
Supplement: Supplementary file 1 [file diagnostics-15-01234-s001.zip › S10.pdf]

Appendix 10. Incidence of adverse events among different procedures (Estimate per 1,000 Procedural Sedations)

Adverse Events	EGDS + Colonoscopy	EUS	ERCP	PEG	Enteroscopy	First level procedures	Second level procedures	p-value
Hypoxia								<0,00001
Events	3.563/483.681	75/927	112/1.436	82/194	3/73	3.563/483.681	426/5.529	
Estimate per 1.000 (‰)	30	98	86	42*	40	26	10*	
95%CI	20-40	37-183	34-156	35-49	3-103	19-33	7-141	
I^2(%)	99,2	91,6	92,7	ND	ND	99	94	
Hypotension								0,59
Events	4.027/140.110	8/927	85/1.436	2/47	5/73	4.027/140.110	245/5.382	
Estimate per 1.000 (‰)	40	10	85	42	68	42	49	
95%CI	20-70	0,1-42	18-190	12-142	18-140	22-67	25-80	
I^2(%)	99,7	87,0	96,5	ND	ND	99	94	
Bradicardia								0,08
Events	1.387/289.855	19/927	45/1.436	0/47	2/73	1.387/289.855	71/3.052	
Estimate per 1.000 (‰)	10	28	34	0	18	10	21	
95%CI	0,5-20	0,1-89	4-86		0,1-69	5-16	6-40	
I^2(%)	99,2	92,6	92,8	ND	ND	99	87	
Major								0,0015
Events	8/204.760	1/927	1/1.937	0/47	0/73	36/482.141	13/5.883	
Estimate per 1.000 (‰)	0,03	0,001	0,01	0	0	0,1	3,1	
95%CI	0,01-14	0,001-2,7	0,001-1,1			0,001-0,2	1,3-5,0	
I^2(%)	67,8	0	0	ND	ND	74	0	

* reported on 100 cases. Results are presented as number of events over the total of patients (only studies that reported the events), estimate on 1.000 patients, 95% Confidence interval and heterogeneity index (I^2); p-value calculated between first level vs second level procedures only
