# Supplementary material for: The Incidence of Adverse Events in Adults Undergoing Procedural Sedation with Propofol Administered by Non-Anesthetists: A Systematic Review and Meta-Analysis
Source: Diagnostics (Basel). 2025 May 14;15(10):1234. doi: 10.3390/diagnostics15101234 (PMC12110594; doi:10.3390/diagnostics15101234)
Supplement: Supplementary file 1 [file diagnostics-15-01234-s001.zip › S11.pdf]

Appendix 11. Sensitivity analysis, events rates in RCT studies and prospective/retrospective studies, not randomized (estimate per 1,000 Procedural Sedations)

Adverse Events	RCT studies	Prosp/Retrosp	p-value
Hypoxia<br>Events<br>Estimate per 1.000 (%)<br>95%CI<br>I^2 (%)	 593/32.156<br>84<br>45-134<br>97	 4.508/907.674<br>30<br>25-36<br>99	 0.003
Hypotension<br>Events<br>Estimate per 1.000 (%)<br>95%CI<br>I^2 (%)	 148/31.199<br>36<br>14-65<br>95	 5.181/538.307<br>40<br>27-55<br>99	 0.937
Bradicardia<br>Events<br>Estimate per 1.000 (%)<br>95%CI<br>I^2 (%)	 97/31.136<br>22<br>6-46<br>94	 1.489/610.798<br>7<br>4-11<br>99	 0.039
Major<br>Events<br>Estimate per 1.000 (%)<br>95%CI<br>I^2 (%)	 99/865.461<br>0,03<br>0,001-0,103<br>0	 108/910.093<br>0,06<br>0,02-0,10<br>48	 0.493

Results are presented as number of events over the total of patients (only studies that reported the events),estimate per 1.000 patients, 95% Confidence interval and heterogeneity index (I^2);
